# A Novel Strategy for Topical Administration by Combining Chitosan Hydrogel Beads with Nanostructured Lipid Carriers: Preparation, Characterization, and Evaluation

**DOI:** 10.3390/gels10030160

**Published:** 2024-02-21

**Authors:** Rui Sun, Qiang Xia, Yufeng Sun

**Affiliations:** 1 Department of Pathology, Medical School of Nantong University, Nantong 226001, China; sunrui@ntu.edu.cn; 2 School of Biological Science and Medical Engineering, State Key Laboratory of Bioelectronics, Southeast University, Nanjing 210096, China

**Keywords:** topical administration, chitosan hydrogel beads, nanostructured lipid carriers, stability, skin permeation

## Abstract

The objective of the present study was to develop and evaluate NLC–chitosan hydrogel beads for topical administration. The feasibility of the preparation technology was verified by investigating various formulation factors and the impact of chitosan hydrogel beads on the NLC. The encapsulation efficiency of NLC–chitosan hydrogel beads was above 95% in optimized process conditions. The physical characterization of the NLC–chitosan hydrogel beads showed that the NLC was distributed within the network of the chitosan hydrogel beads. Furthermore, the incorporation of NLC into the chitosan hydrogel beads was related to the electrostatic interaction between the surface of the NLC and chitosan, which influenced the lipid ordering degree of the NLC and contributed to the stability. The stability studies showed that the retention rate of quercetin in the NLC–chitosan hydrogel beads was 88.63 ± 2.57% after 10 months of storage under natural daylight. An in vitro permeation study showed that NLC–chitosan hydrogel beads exhibited superior ability in enhancing skin permeation by hydrophobic active ingredients compared to the NLC and significantly increased skin accumulation. These studies demonstrated that the use of NLC–chitosan hydrogel beads might be a promising strategy for the delivery of hydrophobic active ingredients in topical administration.

## 1. Introduction

Topical administration, a non-invasive drug delivery method, offers a distinct advantage by enabling the targeted delivery of higher active ingredient doses while minimizing systemic exposure [1]. Lipid-based nanocarriers, including liposomes, emulsions, and lipid nanoparticles, are commonly utilized in topical administration due to their ideal permeability and low toxicity [2,3]. At the end of the last century, lipid nanoparticles were developed as alternatives to other systems in cosmetic and dermatological formulations [4]. Compared to conventional lipid-based nanocarriers, lipid nanoparticles offer several advantages, including avoiding organic solvents during preparation and facile industrial scalability [5]. Otherwise, lipid nanoparticles can form a compact film on the skin surface due to their high crystallinity, thereby increasing skin hydration and promoting the permeation of active ingredients [6]. Consequently, lipid nanoparticles exhibit more potential in transdermal delivery than conventional lipid-based nanocarriers. The first generation of lipid nanoparticles, known as solid lipid nanoparticles (SLN), were formulated by substituting liquid lipids in traditional water-in-oil nanoemulsions with solid lipids [5]. To tackle the challenges associated with SLN application, a new generation of lipid nanoparticles, referred to as nanostructured lipid carriers (NLC), was also developed from oil-in-water emulsion [7]. This novel type of lipid nanoparticle incorporates a combination of solid and liquid lipids rather than a single solid lipid, thereby effectively reducing the melting point of the lipid matrix (still above body temperature). The incorporation of liquid lipids disrupts the ordered structure of the lipid matrix, resulting in an increased number of lattice defects. Consequently, the NLC is referred to as nanostructured. These lattice defects provide additional space to enhance the loading capacity for hydrophobic active ingredients, preventing expulsion during storage [8]. Therefore, NLCs inherit all the advantages of SLN and demonstrates superior loading capacity for active ingredients and controlled release properties.

However, two challenges still exist associated with the topical administration of NLC. First, due to the reconfiguration of the crystal structure, the shape and surface area of the NLC might be changed during storage, leading to the aggregation of the NLC. In addition, the high external aqueous phase in lipid-based nanocarrier systems leads to low viscosity, resulting in inadequate spreadability and retention on the skin surface. Therefore, the semi-solidification of lipid-based nanocarrier systems is essential for practical application [9]. Currently, there are two primary strategies for the semi-solidification of lipid-based nanocarrier systems. The viscosity could be directly improved by preparing a concentrated dispersion of lipid-based nanocarriers [6]. Another approach combines the dispersion of lipid-based nanocarriers with high-viscosity formulations, such as creams and hydrogels. Among these strategies, hydrogel-based systems are regarded as more promising due to their easy preparation, improved stability, and the absence of greasiness [10,11,12].

Hydrogels are hydrophilic polymer networks formed through crosslinking polymers, which can absorb a large amount of water without dissolving. Hydrogels are commonly employed as a delivery system in the food, pharmaceutical, and cosmetic industries [13,14]. To enhance the performance of emulsion-based delivery systems, emulsion-filled hydrogels, also referred to as emulsion gels, were developed based on hydrogels and have garnered increasing attention over the past ten years [15]. It is an effective strategy to improve the stability of emulsions and enable the hydrogel to encapsulate hydrophobic active ingredients. The low stability of emulsions might be attributed to flocculation and coalescence caused by Brownian motion. By immobilizing the oil droplets within the three-dimensional structure of the hydrogel, Brownian motion is restricted, leading to an increase in stability [16]. Emulsion-filled hydrogels are applied to increase the thermodynamic stability of emulsion and control the release of active ingredients [17]. Otherwise, hydrogel-based systems offer the advantage of skin hydration and adhesion and can even enhance the transdermal permeation of active ingredients [18]. Therefore, emulsion-filled hydrogels have also been used to improve the performance of emulsions, such as microemulsions and nanoemulsions, in topical administration [19,20,21]. NLC is developed based on traditional water-in-oil nanoemulsions using a combination of solid and liquid lipids as the lipid phase. Thus, emulsion-filled hydrogels might be a potential method to address the problems in the topical application of NLC. Combining emulsion-filled hydrogels with NLC makes it possible to enhance the stability and permeation performance of NLC, achieving a system with the advantages of NLC and emulsion-filled hydrogels.

Chitosan is a natural polysaccharide with advantages such as low cost, wide availability, non-toxicity, biodegradability, high biocompatibility, and antibacterial properties [22]. It is worth noting that chitosan exhibits strong adhesive properties and can promote the moisture content of the stratum corneum and the permeation of active ingredients [23,24,25]. Therefore, chitosan is widely used in topical administration. Chitosan can form hydrogel beads through different crosslinking methods, including physical and chemical crosslinking. Tripolyphosphate crosslinking is commonly applied for chitosan gelation due to its non-toxicity and excellent mechanical properties [26]. The amino groups of chitosan interact electrostatically with tripolyphosphate to induce intra- and intermolecular crosslinking of chitosan molecules [27,28].

Quercetin is a natural hydrophobic active ingredient, possessing various biological effects such as antioxidant, anticancer, and anti-inflammatory properties [29]. Despite the numerous advantageous effects of quercetin, its low aqueous solubility (2 μg/mL) poses challenges and leads to limited bioavailability [30]. Hence, addressing these challenges to enhance the therapeutic efficacy of quercetin constitutes a primary focal point within the realms of the food, pharmaceutical, and cosmetic industries. The present study aimed to assess the feasibility of NLC–chitosan hydrogel beads as a novel strategy for topical administration. Quercetin was used as a model hydrophobic active ingredient. This novel chitosan-based delivery system was developed by combining filled hydrogel beads with nanostructured lipid carriers to integrate their advantages. Chitosan hydrogel beads might be used to enhance the stability of nanostructured lipid carriers loaded with hydrophobic active ingredients, providing a feasible approach for semi-solidifying nanostructured lipid carrier systems. The integration of nanostructured lipid carriers and chitosan hydrogel beads might exhibit a potential enhancement in the skin permeation of hydrophobic active ingredients, thereby presenting an innovative approach for topical administration and facilitating the broader application of chitosan-based delivery systems.

## 2. Result and Discussion

### 2.1. Fabrication of Quercetin-Loaded NLC

In the present study, a quercetin-loaded NLC was prepared using a high-pressure homogenization technique. The selection of matrix lipids should be conducted with great care, considering their significant impact on the physical characterizations of NLC [5]. The lipid screening was based on the solubility of quercetin in lipids and the compatibility of quercetin with lipids. For liquid lipids, the highest solubility of resveratrol was found in octyl decyl glycerate (ODO) (Table 1). ODO is a medium-chain triglyceride (MCT) and is widely used in NLC preparation [31]. Although the solubility of quercetin in stearic acid was found to be higher than that in glycerol monostearate (GMS), the compatibility between quercetin and stearic acid was poor (Table 2). Therefore, GMS was selected as the solid lipid for NLC preparation. In conclusion, NLC was prepared using a mixture of ODO and GMS as the lipid matrix.

The particle size, PDI, and zeta potential of NLC are shown in Table 3. To understand the characteristics of the system, an empty NLC (without quercetin) was prepared. The mean particle size and PDI of the empty NLC (without quercetin) were determined to be 74.4 ± 1.1 nm and 0.209 ± 0.009, respectively. The low PDI value indicated a narrow distribution of dispersed NLC. The zeta potential of the empty NLC was −41.2 ± 1.3 mV. According to the literature, a zeta potential higher than 30.0 mV is considered to be stable [32]. The mean particle size and PDI of the quercetin-loaded NLC dispersion were determined to be 77.8 ± 2.7 nm and 0.168 ± 0.017, respectively. It was found that, after loading quercetin into the lipid nanoparticles, there was a slight increase in the particle size. In addition, the loading of quercetin also affected the surface charge of the NLC. These results might be related to the quercetin enriched on the surface of the NLC.

### 2.2. Fabrication of NLC–Chitosan Hydrogel Beads

**Figure 1:**
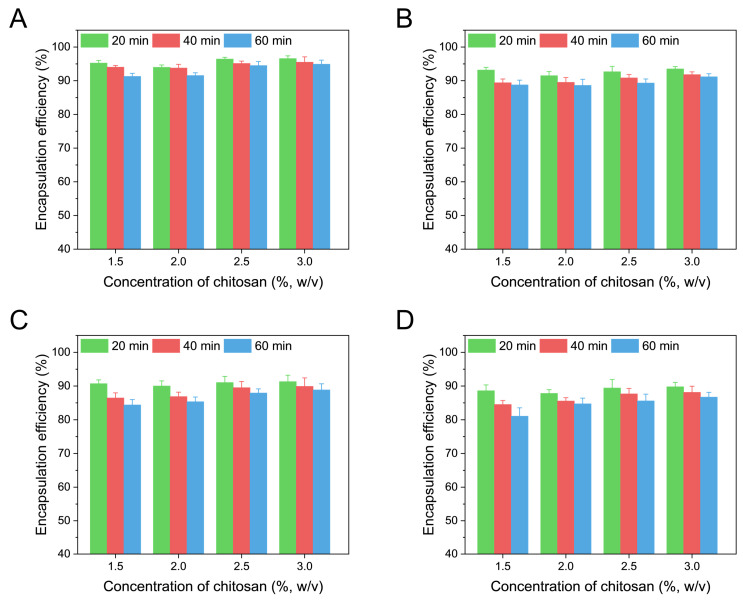
Effects of chitosan concentration and crosslinking time on encapsulation efficiency of NLC–chitosan hydrogel beads at different sodium tripolyphosphate concentrations in crosslinking solution: (**A**) sodium tripolyphosphate concentration at 0.5% (*w*/*v*), (**B**) sodium tripolyphosphate concentration at 1.5% (*w*/*v*), (**C**) sodium tripolyphosphate concentration at 2.5% (*w*/*v*), (**D**) sodium tripolyphosphate concentration at 3.5% (*w*/*v*).

In the present study, the NLC was prepared using a high-pressure homogenization technique and then incorporated into the chitosan hydrogel through ionic gelation. During the preparation of the NLC, soybean lecithin was used as the co-emulsifier, which could generate negative charges on the surface of NLC and thus facilitate the formation of NLC–chitosan hydrogel beads through electrostatic adsorption. The encapsulation efficiency variation between the NLC and NLC–chitosan hydrogel beads was applied to evaluate the preparation technology of the NLC–chitosan hydrogel beads. The encapsulation efficiency of the model active ingredients in the initial NLC was measured to be 97.17 ± 0.39%. As shown in Figure 1, the encapsulation efficiencies of all NLC–chitosan hydrogel beads were found to be lower than that of the initial NLC. This result might suggest that, besides free quercetin in the aqueous phase of the NLC dispersion, quercetin encapsulated in NLC also escaped from the chitosan hydrogel beads during the crosslinking process. Both the chitosan concentration and sodium tripolyphosphate concentration significantly impacted the encapsulation efficiency of the NLC–chitosan hydrogel beads. With the increase in chitosan concentration, the leakage during the crosslinking process was decreased. It is worth noting that by increasing the sodium tripolyphosphate concentration, the encapsulation efficiency of the NLC–chitosan hydrogel beads was decreased. This result indicated that the incorporation of NLC in beads could be attributed to both spatial hindrance and electrostatic adsorption, which arise from the electrostatic interaction between the phospholipid on the surface of the NLC and the amino group of the chitosan network. Tripolyphosphate also interacts electrostatically with the amino groups of chitosan to induce crosslinking of chitosan molecules. Therefore, the high concentration of tripolyphosphate might decrease the amount of NLC-binding sites on the beads, thereby hindering incorporation and leading to leakage. When the concentration of chitosan was high (2.5% and 3%, *w*/*v*) while maintaining a low concentration of sodium tripolyphosphate (0.5%, *w*/*v*), the NLC–chitosan hydrogel beads exhibited a higher encapsulation efficiency (>95%), indicating that the preparation technology used could achieve NLC–chitosan hydrogel beads with excellent encapsulation capability.

### 2.3. Influence of Chitosan Hydrogel Beads on NLC

#### 2.3.1. Zeta Potential Analysis

**Figure 2:**
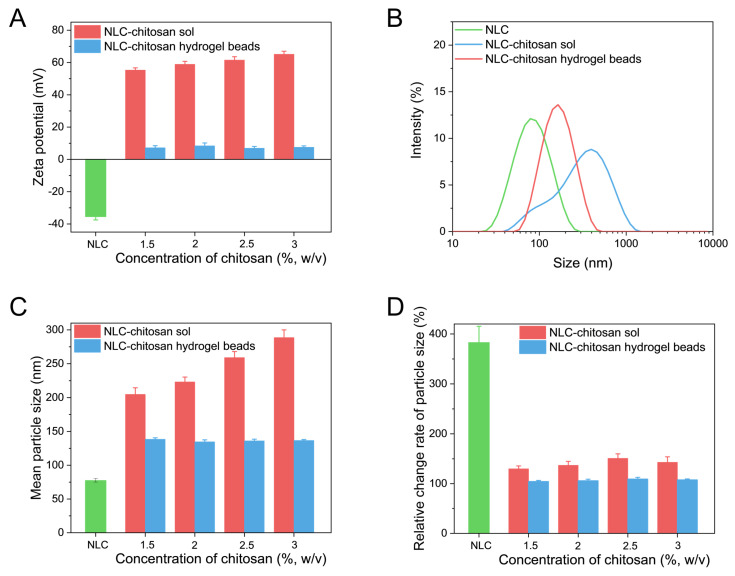
(**A**) Zeta potential of NLC, NLC in chitosan sol, and NLC released from chitosan hydrogel beads. (**B**) Size distribution of NLC, NLC in chitosan sol (1.5% (*w*/*v*) of chitosan), and NLC released from chitosan hydrogel beads (1.5% (*w*/*v*) of chitosan). (**C**) Mean particle size of NLC, NLC in chitosan sol, and NLC released from chitosan hydrogel beads. (**D**) The relative change rate of the mean particle size of NLC, NLC in chitosan sol, and NLC in chitosan hydrogel beads after storage under conditions of 40 °C for 18 days.

The zeta potential was applied to characterize the surface charge of the NLC (Figure 2A). In the present study, negatively charged phospholipids were used as co-emulsifiers to stabilize the NLC and form the negatively charged surface. The zeta potential of the initial NLC was measured to be approximately −35 mV, indicating that the initial NLC was highly negatively charged. After being mixed with chitosan, the NLC in sol was transformed from negatively charged to highly positively charged, and the increase in chitosan concentration led to an enhancement in the zeta potential. This is due to the fact that chitosan was adsorbed onto the surface of NLC. The negatively charged phospholipid on the surface of the NLC might interact with amine groups of chitosan, resulting in the formation of chitosan-coated NLC in sol [33]. After the crosslinking, the NLC was recycled from the chitosan hydrogel beads and analyzed to study the effect of chitosan on the zeta potential of NLC. The positively charged surface of the recycled NLC might also indicate the deposition of chitosan onto the surface of the NLC. The difference between different systems in zeta potential might be due to different thicknesses of chitosan deposition.

#### 2.3.2. Particle Size Analysis

The size distribution and mean particle size of NLC in different systems are shown in Figure 2B,C. The particle size of NLC in chitosan sol exhibited a significant increase compared to the initial NLC and displayed a broader size distribution. Moreover, the size of NLC in chitosan sol was increased with the increase in chitosan concentration. This result might be attributed to the larger self-assembled aggregates and the thicker coating layer caused by the increased chitosan concentration [34]. Otherwise, due to the highly attractive depletion force, the free chitosan in the aqueous phase could cause the depletion flocculation of NLC, leading to an increase in size. Similarly, the mean size of the recycled NLC was larger than that of the initial NLC. This result suggested that, although chitosan was crosslinked by tripolyphosphate to form a chitosan network, the deposition of chitosan onto the surface of the NLC still occurred. The particle size of the NLC recycled from chitosan hydrogel beads was lower than that of the NLC in chitosan sol. Additionally, it was observed that varying the chitosan concentration had no significant impact on the particle size of the recycled NLC, suggesting that chitosan concentration had no influence on chitosan deposition on the surface of NLC in the formation of beads. This phenomenon might be related to multiple layers of chitosan adsorbed onto nanoparticle surfaces in chitosan sol. The outer layers were more prone to detachment during the formation of beads, while inner layers exhibited greater stability and tightness. This adsorption effect depended on the properties of the nanoparticles and polymers rather than polymer concentration [35,36].

To further study the influence of chitosan on the particle size and physical stability of the NLC, all samples were kept in glass vials at the temperature of 40 °C and measured after 18 days of storage. The relative change rate in particle size is shown in Figure 2D, which was the ratio of the mean size after storage to the original mean size. The particle size of the NLC showed a significant increase after storage. The increase in size was essentially related to Brownian motion, which resulted in the collision and aggregation of the NLC. The chitosan sol could significantly reduce the size increase in the NLC. This might be due to the higher electrostatic repulsion caused by the deposition of chitosan onto the surface of the NLC. However, a significant increase was still observed in the particle size of NLC–chitosan sol during storage. The free chitosan in the aqueous phase could cause the aggregation of NLC through the attractive depletion force, which hence promoted the increase in size [35]. Except for the aggregation of nanoparticles, the deposition of chitosan onto the surface of nanoparticles during storage might also increase the particle size [34]. The relative size change rate in the NLC from chitosan hydrogel beads was less than 110%, indicating that chitosan hydrogel beads were more effective in enhancing the stability of NLC. This stabilization effect was related to the elimination of free chitosan and the network of hydrogel beads. As the free chitosan was crosslinked by tripolyphosphate, the attractive depletion force and the continuous deposition of chitosan onto the NLC were prevented during storage. Additionally, the hydrogel network restricted the mobility of the rigid NLC, thereby reducing the possibility of the collision of nanoparticles.

### 2.4. Physical Characterizations of NLC–Chitosan Hydrogel Beads

#### 2.4.1. Scanning Electron Microscopy (SEM) Analysis

**Figure 3:**
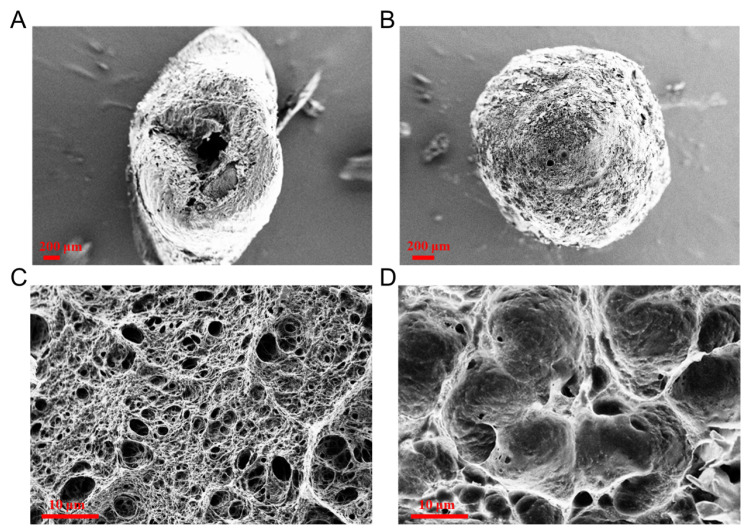
SEM images of surface morphology: blank chitosan hydrogel beads (**A**) and NLC–chitosan hydrogel beads (**B**). SEM images of cross-sections: blank chitosan hydrogel beads (**C**) and NLC–chitosan hydrogel beads (**D**).

The surface morphology and cross-sections of blank chitosan hydrogel beads and NLC–chitosan hydrogel beads were obtained through SEM to investigate the microstructure of filled chitosan hydrogel beads. The overall structure of the blank chitosan hydrogel beads was shrunken with an apparent cavity after dehydration. In contrast, the NLC–chitosan hydrogel beads exhibited a relatively regular spherical shape without significant collapse (Figure 3A,B). This difference might be because insoluble solid-state NLC could fill the pores of chitosan hydrogel beads, thereby reinforcing their structure. It could be observed that the blank chitosan hydrogel beads exhibited a loose microstructure with many tiny voids (Figure 3C). When the NLC was incorporated into the chitosan hydrogel, there were relatively fewer voids inside the chitosan network, indicating the uniform distribution of NLC within the chitosan hydrogel beads (Figure 3D). Otherwise, the decrease in void fraction caused by the incorporation also suggested the distribution of NLC within chitosan hydrogel beads (Table S1).

#### 2.4.2. Attenuated Total Reflectance-Fourier Transform Infrared Spectroscopy (ATR-FTIR) Analysis

**Figure 4:**
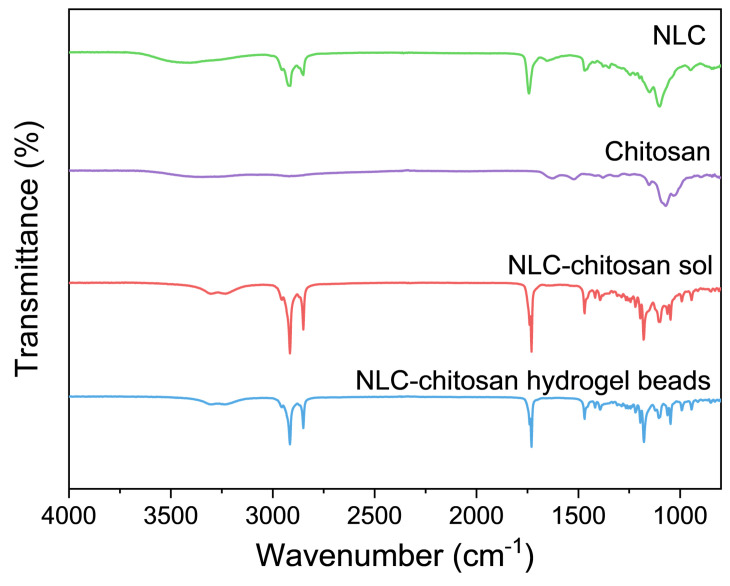
ATR-FTIR spectra of NLC, chitosan, NLC–chitosan sol, and NLC–chitosan hydrogel beads.

In order to confirm the incorporation mechanism, the interaction between the NLC and chitosan was investigated through ATR-FTIR analysis. The infrared spectra for NLC, chitosan, NLC–chitosan sol, and NLC–chitosan hydrogel beads are shown in Figure 4. The NLC exhibited characteristic infrared absorption peaks at 2915 cm^−1^ (C–H stretching vibration), 2850 cm^−1^ (C–H stretching vibration), and 1740 cm^−1^ (C=O stretching vibration). The spectrum of chitosan displayed characteristic infrared absorption peaks at 1628 cm^−1^ (amide C=O stretching vibration) and 1523 cm^−1^ (N-H bending vibration). The characteristic infrared absorption peaks of chitosan at 1628 cm^−1^ and 1532 cm^−1^ almost disappeared in the infrared spectrum of the NLC–chitosan sol. Otherwise, the characteristic infrared peak of NLC at around 1740 cm^−1^ shifted to a lower wavenumber range. These results indicated an electrostatic interaction between chitosan and phospholipids on the surface of NLC [37,38]. The characteristic absorption peaks of the NLC–chitosan hydrogel beads were similar to those of the NLC–chitosan sol, suggesting that the NLC might be incorporated into chitosan hydrogel beads through an electrostatic interaction. Because of the large pore size of chitosan hydrogel beads, this interaction was meaningful for the incorporation of the NLC into the chitosan hydrogel beads.

#### 2.4.3. X-ray Diffraction (XRD) Analysis

**Figure 5:**
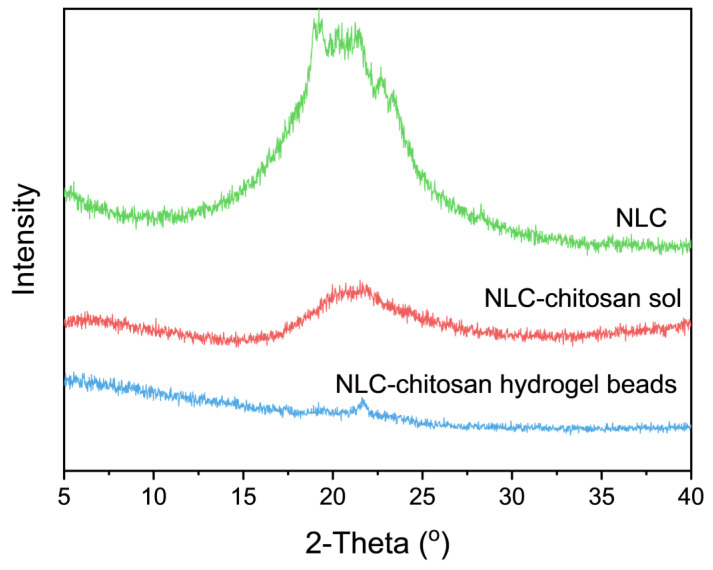
XRD patterns of NLC, NLC–chitosan sol, and NLC–chitosan hydrogel beads.

XRD analysis was conducted to study the influence of chitosan on the polymorphism of the NLC. The XRD patterns of the NLC, NLC–chitosan sol, and NLC–chitosan hydrogel beads are shown in Figure 5. It could be observed that there was no occurrence of supercooled lipid matrix in these samples. The diffraction peak observed at a 2θ diffraction angle of 21.8° for NLC indicated the presence of an α-form crystal structure in the solid lipid matrix, which exhibited a relatively disordered crystalline arrangement and thus facilitated the encapsulation of hydrophobic active ingredients. The diffraction peak at around 21.8° in the XRD pattern of NLC–chitosan sol indicated that the α-form crystal structure still existed in the lipid matrix of NLC. Similarly, the diffraction peak at 21.8° was also observed in the XRD pattern of the NLC–chitosan hydrogel beads, suggesting an α-form crystal structure in the incorporated NLC. The results from the XRD analysis revealed that the microstructure of the lipid matrix remained relatively disordered after incorporating the NLC into chitosan hydrogel beads.

#### 2.4.4. Differential Scanning Calorimetry (DSC) Analysis

**Figure 6:**
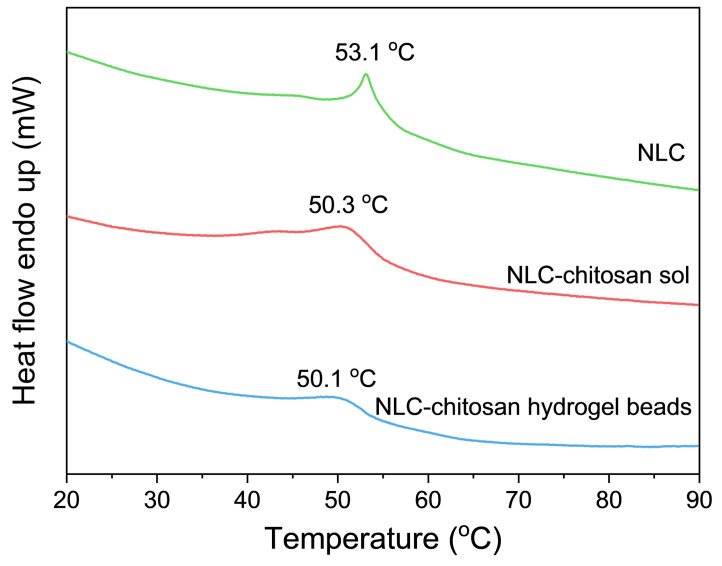
DSC thermograms of NLC, NLC–chitosan sol, and NLC–chitosan hydrogel beads.

DSC was used to analyze the state of the lipid matrix to elucidate the influence of chitosan on the NLC. The DSC thermograms of the NLC, NLC–chitosan sol, and NLC–chitosan hydrogel beads are presented in Figure 6. The thermograms of NLC exhibited a sharp endothermic peak at 53.1 °C, indicating that the lipid matrix of the initial NLC was in a solid state [31,39]. The endothermic peak of the NLC–chitosan sol was broadened and shifted to 50.3 °C, lower than the NLC. This phenomenon was because chitosan coating might influence the lipid matrix of the NLC and lead to a looser and more disordered lipid matrix [40]. The formation of a more disordered crystalline structure in the lipid matrix was beneficial for encapsulating hydrophobic active ingredients within the lipid nanoparticles. Instead, an ordered lipid modification tended to extrude encapsulated hydrophobic active ingredients [41]. For NLC–chitosan hydrogel beads, the endothermic peak of the lipid matrix was also broadened and shifted to a lower temperature. This result suggested that the chitosan network of hydrogel beads affected the ordering degree of the lipid matrix, which could enhance their ability to encapsulate hydrophobic active ingredients.

### 2.5. Chemical Stability Study

**Figure 7:**
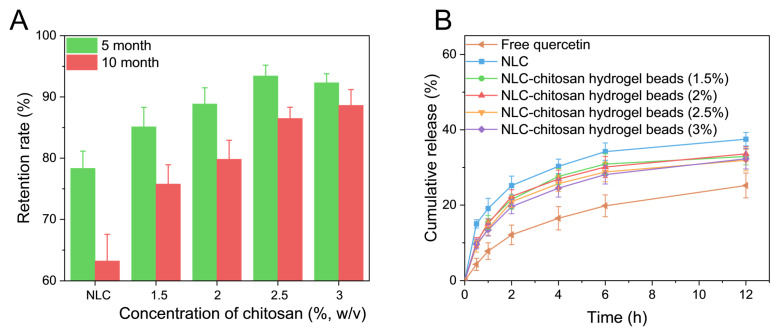
(**A**) Chemical stability of quercetin in NLC and NLC–chitosan hydrogel beads (at different chitosan concentrations) exposed to natural light during 10 months of storage. (**B**) In vitro release profiles of free quercetin and quercetin from NLC and NLC–chitosan hydrogel beads (at different chitosan concentrations).

The chemical stability of quercetin in NLC–chitosan hydrogel beads prepared with different chitosan concentrations was investigated and compared with the NLC sample (Figure 7A). The NLC is regarded as a promising delivery system for enhancing the chemical stability of susceptible hydrophobic active ingredients. However, after 10 months of storage, the retention of quercetin in the NLC was only 63.2%. Compared to the NLC, the stability of quercetin in the NLC–chitosan hydrogel beads was significantly increased. Otherwise, the stability of quercetin in chitosan hydrogel beads exhibited an upward trend with the increase in chitosan concentration. The results indicated that incorporating an NLC into chitosan hydrogel beads could enhance the chemical stability of hydrophobic active compounds encapsulated in the NLC during storage. This stabilization effect could be attributed to the high physical stability and low lipid ordering degree of the NLC incorporated into chitosan hydrogel beads.

### 2.6. In Vitro Release Study

An in vitro release study of quercetin from different systems was conducted using the dialysis bag method. The in vitro cumulative release profiles of free quercetin, NLC, and NLC–chitosan hydrogel beads prepared with different chitosan concentrations are shown in Figure 7B. The cumulative release rate of quercetin from the NLC and NLC–chitosan hydrogel beads was significantly higher than that of quercetin powder. This might be due to the fact that quercetin was molecularly dispersed in the NLC, which provided a larger surface area of quercetin available for dissolution [42]. Moreover, the faster release rate of quercetin from the NLC systems during the first 30 min might be related to the distribution of quercetin on the outer layer of the NLC. After 12 h, approximately 38% of the quercetin was released from the NLC, indicating the majority of active ingredients were encapsulated within the semisolid core of the NLC. It could be observed that the chitosan concentration had no significant influence on the cumulative release amount. The cumulative release profiles of quercetin from the NLC–chitosan hydrogel beads were similar to that of the NLC, with a fast release rate observed in the initial stage, suggesting that part of the quercetin still resided on the outer layer of the NLC after incorporation. The cumulative release amount of quercetin from NLC–chitosan hydrogel beads was slightly lower than that from the initial NLC. This phenomenon was attributed to the network of hydrogel beads, which could influence the diffusion of nanoparticles and consequently impede release.

### 2.7. In Vitro Skin Permeation Study

**Figure 8:**
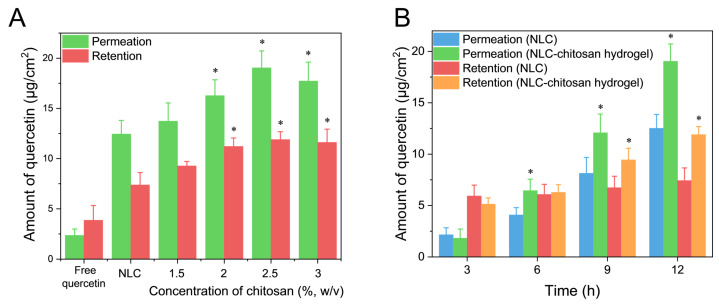
(**A**) The amount of quercetin penetrating through the skin and accumulating in the skin from free quercetin aqueous dispersion, NLC, and NLC–chitosan hydrogel beads (at different chitosan concentrations) after 12 h of treatment. (**B**) The amount of quercetin penetrating through the skin and accumulating in the skin from NLC and NLC–chitosan hydrogel beads (2.5% (*w*/*v*) of chitosan) after 3 h, 6 h, 9 h, and 12 h of treatment. * Statistically significant differences from NLC (*p* < 0.05).

The transdermal permeation and skin accumulation of quercetin from the quercetin aqueous dispersion, NLC, and NLC–chitosan hydrogel beads prepared with different chitosan concentrations after 12 h of treatment are illustrated in Figure 8A. The amount of quercetin that penetrated through the skin from the NLC was measured to be 12.45 ± 1.35 μg/cm^2^, which was significantly higher than that from the aqueous dispersion (2.23 ± 0.64 μg/cm^2^). Meanwhile, the skin accumulation amount of quercetin from the NLC (7.38 ± 1.23 μg/cm^2^) was significantly higher than that from the aqueous solution (3.87 ± 1.46 μg/cm^2^). These results demonstrated that the utilization of NLC effectively enhanced the permeation of quercetin and promoted its accumulation in the skin. This improved permeation could be attributed to the occlusion effect of the NLC, which improved the moisture content of the stratum corneum and consequently influenced its barrier function [6].

Except for the NLC–chitosan hydrogel beads prepared with a chitosan concentration of 1.5% (*w*/*v*), the transdermal permeation and skin accumulation amounts of quercetin from NLC–chitosan hydrogel beads after 12 h of treatment were significantly higher than those from the NLC. The present findings indicated that the NLC–chitosan hydrogel beads had no adverse effect on the permeation of hydrophobic active ingredients and even enhanced permeation. However, this advantage correlated with the concentration of chitosan used in the preparation. It is widely acknowledged that the barrier function of the stratum corneum hinders transdermal penetration of hydrophobic active ingredients [43]. The observed increase in permeation facilitated by NLC–chitosan hydrogel beads might be attributed to their effect on the barrier function of the stratum corneum. The utilization of high-crystallinity lipid nanoparticles in combination with semi-solid hydrogels effectively enhanced the moisture content of the stratum corneum and thus disturbed its barrier function [6,44]. Otherwise, chitosan might influence the barrier function of the stratum corneum by modulating its structural integrity [45]. Moreover, semi-solid hydrogels demonstrated good adhesive properties on the skin surface, facilitating the permeation of active ingredients.

The effect of treatment time on skin permeation by quercetin from different systems was studied using chitosan hydrogel beads prepared with a chitosan concentration of 2.5% (*w*/*v*). The results are shown in Figure 8B and Table S2. The flux value of quercetin from NLC–chitosan hydrogel beads (2.078 ± 0.106 μg/cm^2^/h) was significantly higher than that from NLC (1.400 ± 0.024 μg/cm^2^/h). The significant difference in permeation amount emerged after 6 h of treatment, and the significant difference in skin accumulation amount appeared after 9 h of treatment. As the treatment duration increased, the difference in transdermal permeation between the two groups gradually increased. The results suggested that the penetration enhancement by chitosan hydrogel beads was dependent on treatment time.

### 2.8. Permeation Pathway Study Based on Hydrophobic Fluorescent Probe

To further elucidate the permeation mechanism of NLC–chitosan hydrogel beads, Nile red, a hydrophobic fluorescent probe, was encapsulated into the lipid phase of NLC. Nile red has been extensively employed to investigate the permeation of lipid-based nanocarriers, as it can visually demonstrate the permeation profile [46,47,48,49,50]. It is worth noting that the distribution of Nile red and quercetin in the skin might not be identical due to their distinct physicochemical properties, including solubility and logP. Nonetheless, this visualization method offers advantages in elucidating the permeation profile of the delivery system and can be employed to investigate the permeation pathway.

**Figure 9:**
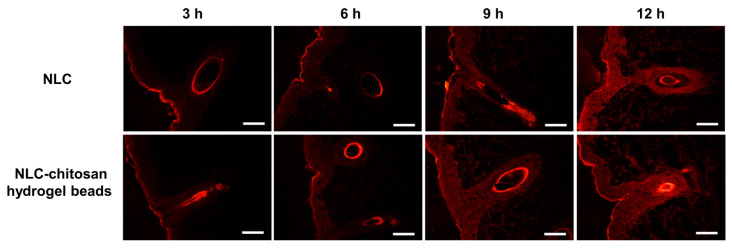
Cross-section fluorescence microscopy of the skin treated with NLC and NLC–chitosan hydrogel beads (2.5% (*w*/*v*) of chitosan) after 3 h, 6 h, 9 h, and 12 h of treatment. Scale bars, 100 μm.

The results are shown in Figure 9 and Table 4. The fluorescent intensity of the skin treated with the NLC–chitosan hydrogel beads was significantly higher than that of the skin treated with the NLC after 6 h of treatment. This result was consistent with the permeation of quercetin and further demonstrated the role of NLC in facilitating the permeation of hydrophobic active ingredients encapsulated in NLC. Additionally, the result of fluorescent intensity also indicated that the enhancement in penetration emerged after 6 h of treatment, suggesting that the penetration enhancement was dependent on treatment time. Similar to the NLC, the NLC–chitosan hydrogel beads effectively promoted the permeation of Nile red into the skin through two pathways, including hair follicles and the stratum corneum. The findings indicated that incorporating the NLC into the chitosan hydrogel had no significant effect on the permeation pathway of the hydrophobic active ingredients encapsulated in the NLC. After 3 h of treatment, the skin accumulation of Nile red was mainly located in the hair follicles, suggesting that Nile Red could penetrate the skin via hair follicles. Due to its rigid structure, the NLC could not directly pass through the stratum corneum into the skin in the early stage. However, it could still deliver hydrophobic active ingredients into the skin via the hair follicles [51]. Hair follicles play an essential role in facilitating the permeation of hydrophobic active ingredients encapsulated in NLC delivery systems in the early stage of permeation [52]. The accumulation of Nile red in the stratum corneum gradually increased, and Nile red gradually penetrated into deeper layers of skin through the stratum corneum, suggesting that the role of the permeation through the stratum corneum became more evident in the later stage of treatment.

These findings could be used to elucidate the influence of chitosan hydrogel beads on the permeation of model hydrophobic active ingredients encapsulated in the NLC. In the early stage of treatment, the permeation of hydrophobic active ingredients was mainly related to the pathway via hair follicles. Therefore, there was no significant difference in the permeation amount of quercetin between the NLC and NLC–chitosan hydrogel beads within the initial 3 h. However, as the treatment duration increased, the disturbance to the barrier function of the stratum corneum gradually became pronounced. Consequently, the promotional effect of chitosan hydrogel beads on permeation became significant after 6 h of treatment.

## 3. Conclusions

In summary, NLC–chitosan hydrogel beads were successfully fabricated through the extrusion method combined with tripolyphosphate crosslinking. Various formulation factors and the influence of chitosan hydrogel beads on NLC were investigated to confirm the feasibility of the preparation process. The encapsulation efficiency of NLC–chitosan hydrogel beads was above 95% in optimized process conditions. Structural characterization indicated the uniform distribution of NLC within the network of chitosan hydrogel beads. The result of ATR-FTIR confirmed that there was an electrostatic interaction between the phospholipid on the surface of the NLC and the chitosan, which was meaningful for the formation of the NLC–chitosan hydrogel beads. DSC analysis indicated that incorporating NLC into chitosan hydrogel beads decreased the lipid ordering degree of the NLC. Stability studies demonstrated that incorporating NLC into chitosan hydrogel beads effectively improved the physical stability of the NLC and the chemical stability of model hydrophobic active ingredients encapsulated in the NLC. The release of quercetin from NLC–chitosan hydrogel beads showed a delayed profile. Compared with the NLC, the NLC–chitosan hydrogel beads could enhance the permeation of hydrophobic active ingredients and promote the skin accumulation of hydrophobic active ingredients by disturbing the barrier function of the stratum corneum. The incorporation of an NLC into the chitosan hydrogel beads could not alter the penetration pathway of the hydrophobic active ingredients encapsulated in the NLC. Moreover, it is observed that hydrophobic active ingredients could still penetrate skin through both the stratum corneum and hair follicles. The enhancement in penetration by the chitosan hydrogel beads was related to the treatment duration. This study suggested that NLC–chitosan hydrogel beads could be a promising and innovative approach for the topical administration of hydrophobic active ingredients, thereby facilitating the broader application of lipid nanocarriers and chitosan.

## 4. Materials and Methods

### 4.1. Materials

Chitosan hydrochloride (deacetylation degree 91.5%, molecular weight 20,000 Da) was obtained from Zhengzhou Gebeisi Food Additive Co., Ltd. (Henan, China). GMS was supplied by Croda (East Yorkshire, UK). ODO and propylene glycol monostearate were acquired from Henan Zhengtong Food Technology Co., Ltd. (Henan, China). Stearic acid was supplied by Sinopharm Chemical Reagent Co., Ltd. (Shanghai, China). Peanut oil and corn oil were supplied by COFCO Corporation (Beijing, China). Grape seed oil, tea seed oil, and evening primrose oil were supplied by Suzhou Nakang Biotechnology Co., Ltd. (Jiangsu, China). Tween 80 was supplied by Guangzhou Runhua Chemical Co., Ltd. (Guangdong, China). Soybean lecithin was obtained from Meryas Lecithin Co., Ltd. (Beijing, China). Quercetin (99%, *w*/*w*) was purchased from Shaanxi Kangsheng Biotechnology Co., Ltd. (Shaanxi, China). Nile red was purchased from Sigma-Aldrich (St. Louis, MI, USA). The remaining reagents were of analytical reagent grade.

### 4.2. Lipid Screening

Prior to the fabrication of NLC, lipid screening was conducted to assess the suitability of lipids. The solubility of quercetin in lipids was used to screen lipids. For liquid lipids, to obtain the solubility of quercetin, increasing amounts of quercetin were incrementally added into lipids, with magnetic stirring applied at 800 rpm, until saturation. For solid lipids, lipid was heated to 80 °C, and then increasing amounts of quercetin were added into heated lipids with magnetic stirring at 800 rpm until saturation. After cooling from 80 °C to room temperature, the precipitation of quercetin in solid lipids was observed by an optical microscope (DN-10B, Jiangnan Novel, Nanjing, China).

### 4.3. Fabrication of NLC

The hot high-pressure homogenization method was utilized in the present study to prepare the aqueous NLC dispersion [53]. Firstly, quercetin (0.2 g) was dissolved in a mixture of solid lipid (GMS, 5 g), liquid lipid (ODO, 5 g), emulsifier (Tween 80, 5 g), and co-emulsifier (soybean lecithin, 3 g) at a temperature of 80 °C. Subsequently, ultrapure water (81.8 g) heated to 80 °C was added to the mixture and dispersed under at 600 rpm for 10 min at 80 °C. The resulting emulsion was then subjected to high-shear homogenization using a preheated instrument (FA25, FLUKO, Essen, Germany) for 1 min at 10,000 rpm, forming a pre-emulsion. Then, the pre-emulsion was homogenized using a preheated high-pressure homogenizer (FB-110, LiTu, Shanghai, China) at a pressure of 600 bar for five cycles. Finally, the resultant hot nanoemulsions were gradually cooled to room temperature, forming a dispersion containing NLC. Empty NLC dispersion was prepared using the same formulation composition and preparation method, excluding the inclusion of quercetin.

### 4.4. Fabrication of NLC–Chitosan Hydrogel Beads

The NLC dispersion and the chitosan stock solution were mixed at a ratio of 1:1 (*v*/*v*) and stirred at 200 rpm for 5 min, obtaining NLC–chitosan sol. Then, the extrusion method combined with tripolyphosphate crosslinking was employed to fabricate NLC–chitosan hydrogel beads [54]. In brief, the NLC–chitosan sol was introduced dropwise into the sodium tripolyphosphate solution via a syringe pump at a flow rate of 0.3 mL/min while maintaining a fixed distance of 10 cm between the injection needle and the sodium tripolyphosphate solution. The chitosan hydrogel beads were subsequently allowed to undergo crosslinking in sodium tripolyphosphate solution at ambient temperature for a certain period of time. After crosslinking, the sodium tripolyphosphate crosslinking solution was collected.. After that, hydrogel beads were resuspended in purified water. After 30 s, the water was collected. The cleaning process was repeated twice to remove any excess sodium tripolyphosphate crosslinking solution. Finally, the prepared beads were stored in a dry state rather than as an aqueous dispersion. The washing solution was also collected. The mixture of sodium tripolyphosphate crosslinking solution and washing solution was used for the analysis of encapsulation efficiency.

In the study of encapsulation efficiency, the effects of different concentrations of chitosan in NLC–chitosan sol (1.5%, 2%, 2.5%, 3% *w*/*v*), concentrations of sodium tripolyphosphate in crosslinking solution (0.5%, 1.5%, 2.5%, 3.5%, *w*/*v*), and crosslinking time (20, 40, 60 min) were investigated. In other sections of this study, the concentration of sodium tripolyphosphate and crosslinking time were fixed at 0.5% (*w*/*v*) and 20 min, respectively. Unless otherwise specified in the present study, hydrogel beads were prepared with a chitosan concentration of 3% (*w*/*v*).

### 4.5. Encapsulation Efficiency

To determine the encapsulation efficiency of NLC dispersion, free quercetin in the aqueous phase was separated using ultrafiltration centrifugation [55]. A sample of 400 μL of NLC dispersion was transferred to an ultrafiltration centrifuge tube (molecular cut off: 10 kDa, Millipore). The tube was then subjected to centrifugation at 10,000 rpm for 30 min at 4 °C. Subsequently, all filtrate from the lower part of the tube was collected and analyzed for free quercetin amount at a wavelength of 374 nm using UV spectrophotometry (a-1900PC, Puyuan, Shanghai, China). The encapsulation efficiency of NLC was calculated using the following formula:

$$\mathrm{Encapsulation}\;\mathrm{efficiency}\;\mathrm{of}\;\mathrm{NLC}\;\left(\%\right)=(1\;-\;\frac{W_{F}}{W_{T}})\times 100\%$$

where W_T_ represents the total amount of quercetin, and W_F_ is the amount of free quercetin.

By measuring the amount of quercetin leaked from the hydrogel beads during crosslinking, the encapsulation efficiency of NLC–chitosan hydrogel beads could be calculated. After the preparation of the hydrogel beads, the amount of quercetin in both the crosslinking solution and washing water was determined. The encapsulation efficiency of NLC–chitosan hydrogel beads was then calculated using the following formula:

$$\mathrm{Encapsulation}\;\mathrm{efficiency}\;\mathrm{of}\;\mathrm{NLC}–\mathrm{chitosan}\;\mathrm{hydrogel}\;\mathrm{beads}\;(\%)=(1\;-\;\frac{W_{M}}{W_{T}})\times 100\%$$

where W_T_ represents the total amount of quercetin, and W_M_ is the amount of quercetin in both crosslinking solution and washing water.

### 4.6. Particle Size and Zeta Potential Analysis

The mean particle size, size distribution, and zeta potential were analyzed using photon correlation spectroscopy (PCS) with a Zetasizer (ZS90, Malvern, UK) [56]. The zeta potential was obtained through the measurement of electrophoretic mobility. The measurement was performed at a scattering angle of 90° under a temperature of 25 °C, and 1.330 and 0.8872 were set as the refractive index and viscosity of ultrapure water at this temperature, respectively. Before measurement, aqueous samples (NLC suspension, NLC–chitosan sol) were diluted with ultrapure water to acquire suitable scattered light intensity. For NLC–chitosan hydrogel beads, NLC was released by dissociating beads in HCl solution (0.1 M). The dispersion was filtered using a sieve (50 μm) to remove debris and diluted 100-fold with ultrapure water. Then, the diluted NLC dispersion was placed in an ultrafiltration centrifugal filter tube (10 kDa, Millipore, Bedford, MA, USA) and centrifuged at 10,000 rpm for 10 min to remove HCl. The NLC pellet in the ultrafiltration centrifugal filter tube was redispersed with ultrapure water.

To study the influence of chitosan on particle size and physical stability of NLC, all samples were kept in glass vials at a temperature of 40 °C and measured after 18 days of storage.

### 4.7. SEM Analysis

Surface morphology and cross-section of blank chitosan hydrogel beads and NLC–chitosan hydrogel beads were observed using a scanning electron microscope (SEM) (Ultra Plus, Zeiss, Oberkochen, Germany). Dried blank chitosan hydrogel beads and NLC–chitosan hydrogel beads were fixed onto a sample stage using double-sided carbon adhesive tape and then observed using SEM.

### 4.8. ATR-FTIR Analysis

The infrared spectra of NLC, NLC–chitosan sol, and NLC–chitosan hydrogel beads were obtained by FTIR spectrometer (Nicolet 6700, Thermo Scientific, Madison, WI, USA) [57]. The scanning range was from 4000 to 800 cm^−1^, with a total of 32 scans.

### 4.9. XRD Analysis

The crystal state of NLC, NLC–chitosan sol, and NLC–chitosan hydrogel beads was evaluated by X-ray powder diffraction (Smartlab, Rigaku, Japan) [58]. The X-ray source was a copper anode (40 kV/30 mA). The scanning angle range was from 5° to 40° with a step size of 0.02° and scanning rate of 5°/min.

### 4.10. DSC Analysis

The thermal behavior of NLC, NLC–chitosan sol, and NLC–chitosan hydrogel beads was investigated using differential scanning calorimetry (DSC) (DSC8000, PerkinElmer, Waltham, MA, USA) [59]. Approximately 4 mg of dried sample was precisely weighed and sealed in an aluminum pan. The heating run was conducted from 5 to 90 °C at a heating rate of 5 °C/min.

### 4.11. Chemical Stability Study

The NLC dispersion and NLC–chitosan hydrogel beads with different chitosan concentrations were placed in glass bottles, sealed with plastic caps and stored at room temperature in the sunlight for 10 months. The total content of quercetin in different samples was measured at selected time intervals.

### 4.12. In Vitro Release Study

The release behavior of NLC and NLC–chitosan hydrogel beads was investigated using the dialysis bag method [60]. Prior to the experiment, the initial NLC dispersion was mixed with an equal volume of purified water to ensure comparability between different samples. The release medium was composed of a mixture of ethanol and purified water (3:7, *v*/*v*) in order to meet the sink concentration. Quercetin powder, diluted NLC dispersion (3 mL), and a certain amount of NLC–chitosan hydrogel beads (formed using an equal volume of initial NLC dispersion) were introduced into dialysis bags (molecular cut off: 12–14 kDa, Viskase, Chicago, IL, USA), which were subsequently immersed in 200 mL of release medium. The release study was conducted at a temperature of 37 °C under continuous stirring at a speed of 200 rpm. At predetermined time intervals, 1 mL of release medium was collected to quantify the concentration of quercetin in the medium, followed by replenishment with an equal volume of preheated release medium.

### 4.13. In Vitro Skin Permeation Study

In the present study, in vitro skin permeation study of NLC and NLC–chitosan hydrogel beads was conducted using the vertical Franz diffusion cell method [61]. The volume of the receiving chamber was 6.5 mL, and the effective diffusion area was 2.8 cm^2^. Porcine ear skin provided by Linxi Jingde Co., Ltd. (Xingtai, Hebei, China) was selected as the model skin. The porcine ear skin used possessed a complete skin structure (including the stratum corneum) with a thickness ranging from 0.8 to 1 mm. The hair was removed using a razor. The hairless skin was cut into pieces and washed twice with physiological saline solution.

The physiological saline solution containing Tween 80 (1%, *w*/*v*) was used as the receiving medium to ensure the sink condition. The receiving chamber was filled with the medium. Then, the treated porcine skin samples were securely positioned between the receiving chamber and the release chamber, ensuring that the stratum corneum faced toward the release chamber. Subsequently, quercetin aqueous dispersion (0.1%, *w*/*w*), diluted NLC dispersion (600 μL), and a certain amount of NLC–chitosan hydrogel beads (formed using an equal volume of NLC dispersion) were added into the release chamber. The samples were gently spread on the skin surface to achieve uniform distribution and complete contact with the skin. During the skin permeation study, the receiving chamber was maintained at a temperature of 32 °C, while the magnetic stirring speed in the receiving chamber was set to 100 rpm. At the predetermined time, the medium from the receiving chamber was collected to quantify the permeation of quercetin through the skin. The skin was removed from the diffusion cell and rinsed with physiological saline solution to remove residual samples on the surface. Subsequently, the skin was cut into small pieces and immersed in ethanol solution. After ultrasonication of skin samples and centrifugation at 10,000 rpm for 10 min, the resulting supernatant was collected to determine the accumulation amount of quercetin in the skin.

### 4.14. Permeation Pathway Study Based on Hydrophobic Fluorescent Probe

During the preparation process, the hydrophobic fluorescent dye Nile red (0.2%, *w*/*w*) was added to the lipid mixture to obtain Nile red-loaded NLC. Subsequently, Nile red-loaded NLC was used to prepare NLC–chitosan hydrogel beads loaded with Nile red. In vitro skin permeation study was conducted according to the procedures outlined in Section 4.13. At selected time intervals, the treated skin was rinsed with physiological saline solution to eliminate any residual samples on the surface. Then, the skin was embedded in an OCT embedding medium for quick freezing and subsequently sliced into sections (10 μm) using a cryostat microtome (CryoStar NX50, Thermo, Madison, WI, USA). Finally, the distribution of Nile red in skin was observed using a fluorescence microscope (Eclipse C1, Nikon, Tokyo, Japan). All samples were observed using the same parameters, and the fluorescence intensity was measured using ImageJ [62].

### 4.15. Statistical Analysis

The measurements in the present study were performed in triplicate, and values are shown as means ± standard deviation. Statistical analysis was conducted using Student’s *t*-test, with a significance level set at *p* < 0.05.

## Figures and Tables

**Table 1:** Liquid lipid screening.

Liquid Lipids	Solubility of Quercetin (mg/g)
Peanut oil	2.1
Octyl decyl glycerate	5.2
Grape seed oil	2.3
Tea seed oil	1.9
Corn oil	2.4
Evening primrose oil	1.8
Olive oil	2.2

**Table 2:** Solid lipid screening.

Solid Lipids	Solubility of Quercetin (mg/g)	Compatibility
Stearic acid	11.3	−
Propylene glycol monostearate	4.4	−
Glycerol monostearate	10.4	+

Compatibility represents the precipitation of quercetin observed by optical microscope after cooling from 80 °C to room temperature (−, precipitation; +, no precipitation).

**Table 3:** Mean particle size, PDI, and zeta potential of NLC (with quercetin) and empty NLC.

	Mean Particle Size (nm)	PDI	Zeta Potential (mV)
NLC (with quercetin)	77.8 ± 2.7	0.168 ± 0.017	−35.6 ± 1.9
Empty NLC	74.4 ± 1.1	0.209 ± 0.009	−41.2 ± 1.3

**Table 4:** Fluorescence intensity of the skin treated with NLC and NLC–chitosan hydrogel beads (2.5% (*w*/*v*) of chitosan) after 3 h, 6 h, 9 h, and 12 h of treatment.

	NLC	NLC–Chitosan Hydrogel Beads
3 h	4.154	4.088
6 h	3.910	5.198
9 h	6.842	9.867
12 h	12.048	15.040

These results were calculated based on Figure 9. Fluorescence intensity was measured by ImageJ (version 1.53t).

## Data Availability

All relevant data are included in the manuscript.

## Supplementary Materials

The following supporting information can be downloaded at: https://www.mdpi.com/article/10.3390/gels10030160/s1, Figure S1: mean particle size of NLC released from chitosan hydrogel beads (3% (*w*/*v*) of chitosan) after 5 months of storage at room temperature; Table S1: the void fraction of blank chitosan hydrogel beads and NLC-chitosan hydrogel beads; Table S2: values of flux of quercetin from NLC and NLC-chitosan hydrogel.
