# Supplementary material for: A Novel Strategy for Topical Administration by Combining Chitosan Hydrogel Beads with Nanostructured Lipid Carriers: Preparation, Characterization, and Evaluation
Source: Gels. 2024 Feb 21;10(3):160. doi: 10.3390/gels10030160 (PMC10970608; doi:10.3390/gels10030160)
Supplement: Supplementary file 1 [file gels-10-00160-s001.zip › gels-2873541-supplementary.pdf]

Supplementary materials

Long-term stability study

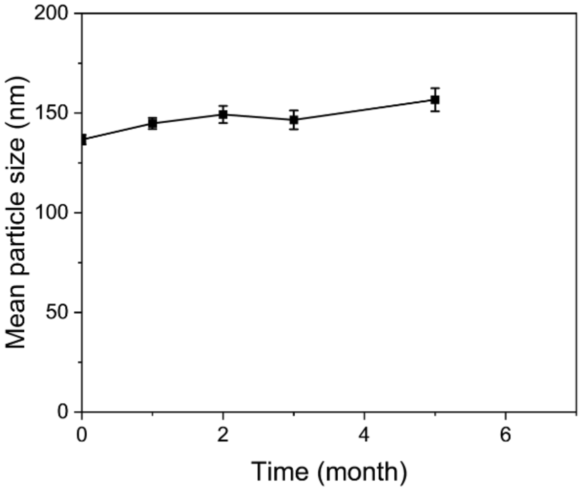

Figure S1. Mean particle size of NLC released from chitosan hydrogel beads (3% (w/v) of chitosan) after 5 months of storage at room temperature.

Void fraction

The blank chitosan hydrogel beads and NLC-chitosan hydrogel beads were dehydrated by the freeze-drying method, and the weight of the dried beads was measured. Then, the void fraction of beads was computed by the following equation:

$$\text{void fraction (\%)}=\frac{V-(M/\rho)}{V}\times 100\%$$

where V is the volume of beads, M is the weight of dried beads, ρ is the density of the mixture of NLC and chitosan.

Table S1. The void fraction of blank chitosan hydrogel beads and NLC-chitosan hydrogel beads

	Void fraction (%)
Blank chitosan hydrogel beads	98.89±0.07
NLC-chitosan hydrogel beads	95.76±0.19

Values of flux

Table S2. Values of flux of quercetin from NLC and NLC-chitosan hydrogel

	Flux (μg/cm^2/h)
NLC	1.400±0.024
NLC-chitosan hydrogel	2.078±0.106

By performing linear regression on the straight portion (6-12 h) of the time-concentration curve for accumulation permeation of quercetin, the slope of the line could be determined as the flux.

Original data of Figure 2C

Chitosan concentration	Mean particle size of NLC in chitosan sol (nm)	Mean particle size of NLC released from chitosan hydrogel beads (nm)
1.5%	204.7±9.8	138.4±2.2
2%	223.2±7.1	134.7±2.9
2.5%	259.1±8.9	136.1±2.5
3%	288.6±11.4	136.7±1.4

Original data of Figure 7A

	Retention rate after 5 month (%)	Retention rate after 10 month (%)
NLC	78.33±2.81	63.24±4.32
NLC-chitosan hydrogel beads (1.5%)	85.12±3.18	75.77±3.14
NLC-chitosan hydrogel beads (2%)	88.86±2.63	79.83±3.09
NLC-chitosan hydrogel beads (2.5%)	93.43±1.76	86.48±1.83
NLC-chitosan hydrogel beads (3%)	92.32±1.47	88.63±2.57

Original data of Figure 8A

	Permeation (μg/cm^2)	Retention (μg/cm^2)
Free quercetin	2.23±0.64	3.87±1.46
NLC	12.45±1.35	7.38±1.23
NLC-chitosan hydrogel beads (1.5%)	13.73±1.81	9.27±0.45
NLC-chitosan hydrogel beads (2%)	16.28±1.57	11.22±0.84
NLC-chitosan hydrogel beads (2.5%)	19.04±1.69	11.90±0.78
NLC-chitosan hydrogel beads (3%)	17.73±1.88	11.62±1.31

Original data of Figure 8B

Time	Permeation (NLC) (μg/cm^2)	Permeation (NLC-chitosan hydrogel) (μg/cm^2)	Retention (NLC) (μg/cm^2)	Retention (NLC-chitosan hydrogel) (μg/cm^2)
3 h	2.16±0.68	1.82±0.90	5.93±1.06	5.13±0.62
6 h	4.08±0.72	6.45±1.11	6.08±0.98	6.28±0.75
9 h	8.13±1.55	12.08±1.82	6.74±1.11	9.44±1.13
12 h	12.52±1.35	19.04±1.69	7.43±1.23	11.9±0.78
